# Post-traumatic cognitive decline and neurodegenerative disease: a narrative review and proposed framework for etiologic assessment

**DOI:** 10.3389/fneur.2026.1844523

**Published:** 2026-10-06

**Authors:** Jae Jin Park, Seung Young Chung, Seong Min Kim, Hyun Dong Yoo

**Affiliations:** Department of Neurosurgery, Eulji University Hospital, Eulji University College of Medicine, Daejeon, Republic of Korea

**Keywords:** Alzheimer’s disease, blood biomarkers, cognitive decline, differential diagnosis, longitudinal assessment, neurodegenerative disease, traumatic brain injury, traumatic encephalopathy syndrome

## Abstract

**Background:**

When a patient with a history of traumatic brain injury (TBI) develops progressive cognitive decline months to years after injury, clinicians must consider injury sequelae, an independent neurodegenerative disease, or both. Existing diagnostic frameworks each address a different aspect of this question, and none was constructed to apportion contribution between them. The distinction matters for management because AD-directed therapy should be considered only after biological Alzheimer’s disease (AD) has been established, followed by separate assessment of treatment eligibility and safety.

**Objective:**

To synthesise evidence on cognitive change after TBI and the clinical, biomarker, and neuroimaging information available when delayed decline is evaluated, and to propose a structure for that evaluation.

**Methods:**

Structured narrative review. A documented *de novo* PubMed search was conducted on 27 July 2026, covering January 2015 to the search date, using two complementary strategies: one addressing cognitive, functional and neurodegenerative outcomes after TBI, and one addressing biomarker and neuroimaging evidence with longitudinal and prognostic terms. Diagnostic and research criteria, neuropathological and mechanistic references, comparator biomarker studies, and treatment trials were identified through targeted retrieval and reference checking. Exhaustive title and abstract screening, dual-reviewer study selection, formal risk-of-bias assessment, and quantitative synthesis were not undertaken.

**Results:**

Four patterns of cognitive change after TBI are proposed as unvalidated longitudinal clinical heuristics rather than validated diagnostic categories, and no prevalence is estimated for any of them. Delayed progressive deterioration following an initial plateau presents the greatest diagnostic difficulty. Blood biomarkers can support evaluation for coexisting biological AD, but neurofilament light and glial fibrillary acidic protein kinetics vary with injury severity and sampling window, and no validated biomarker profile identifies injury-driven neurodegeneration in an individual patient. Tau-related pathology, impaired protein clearance, and chronic neuroinflammatory activity are plausible contributors not shown to form a unified causal cascade.

**Conclusion:**

Current evidence supports structured longitudinal reassessment and evaluation of competing explanations rather than etiological classification by biomarker pattern. A proposed assessment pathway, and a framework for stating what can and cannot be concluded in an individual patient, are offered as testable structures, including for medicolegal settings in which causal attribution is requested.

## 1 Introduction

A patient who sustained a moderate traumatic brain injury (TBI) 2 years earlier attends routine follow-up. Recovery had reached a plateau, but over the preceding 6 months his family has observed new forgetting of recent conversations, disorientation on familiar routes, and a change in the quality of function distinct from the original post-injury deficit. This review addresses the evaluation of delayed or progressive cognitive decline in a patient with a history of TBI: how to assess the possible relationship between the injury and the later decline, and how that assessment informs management.

At the population level, TBI is associated with dementia. Two independent syntheses report elevated risk — a pooled relative risk of 1.66 across 32 studies (1) and a pooled odds ratio of 1.81 (2) — and TBI is among the fourteen modifiable dementia risk factors identified by the Lancet standing Commission (3). Evidence for an association with Alzheimer disease specifically is less consistent. A systematic review and meta-analysis restricted to mild TBI reported a modest increase in future Alzheimer disease risk (pooled RR 1.18, 95% CI 1.11–1.25) (4), whereas a broader meta-analysis pooling heterogeneous TBI studies found no statistically significant association with Alzheimer disease (pooled OR 1.02, 95% CI 0.91–1.15) (2). In the ADNI and DoD-ADNI cohorts, a history of TBI was not associated with Alzheimer biomarker pathology (5). An association between a history of TBI and later dementia does not establish that the injury caused the decline in an individual patient. The likelihood that TBI contributed varies with injury severity, repetitive exposure, age, coexisting disease, and other patient-specific factors. Reverse causality must also be considered: an association concentrated near the time of injury may reflect cognitive or gait impairment that preceded the injury and contributed to it, particularly where pre-injury cognitive information is unavailable.

The question has become more consequential as disease-specific treatment has become available. Anti-amyloid therapies are authorised for early symptomatic Alzheimer disease with confirmed amyloid pathology, so the practical sequence is to establish whether biological Alzheimer disease is present and then to assess treatment eligibility and safety separately. Long-term cognitive follow-up after acute TBI is often fragmented, leaving clinicians without a documented baseline against which later deterioration can be assessed. Individual-level evaluation therefore requires reconstruction of the longitudinal course and assessment of competing explanations rather than inference from the history of TBI alone.

Prior reviews have addressed the population-level mechanisms by which TBI elevates dementia risk (6–8). Recent work in this journal has discussed the bidirectional relationship between TBI and dementia and mapped the bibliometric landscape of post-TBI dementia research (9, 10). Unlike reviews centred on population-level associations or biological mechanisms, this review focuses on individual-level evaluation after delayed cognitive decline and on the implications for disease-specific diagnostic pathways, treatment eligibility, and safety. The sections that follow describe patterns of cognitive change after TBI and the biological pathways proposed to link them, examine the diagnostic frameworks and the clinical, biomarker, and imaging information available for evaluation, and set out a proposed assessment pathway with its management and medicolegal implications.

## 2 Literature search strategy and scope

This structured narrative review was prepared with reference to the SANRA scale for narrative reviews (11). During revision, we conducted a *de novo* PubMed search via PubMed.gov on 27 July 2026, covering records published from 1 January 2015 through the search date. Two complementary strategies were used: a clinical and epidemiological search combining traumatic brain injury and repetitive-head-impact terms with cognitive, functional, dementia, and neurodegenerative outcomes; and a biomarker and neuroimaging search combining traumatic brain injury terms with fluid-biomarker or imaging terms and enriched by longitudinal, prognostic, chronicity, and outcome terms. The searches returned 16,499 and 6,746 records, respectively. Retrieval of 11 prespecified index articles was checked after execution. Ten were retrieved. One biomarker article was not retrieved by either search; this non-retrieval is documented in Supplementary Table S1, illustrating that the searches were used to update and verify coverage of the evidence base rather than to constitute an exhaustive systematic search. The exact queries as entered, PubMed Search Details, execution records, result counts, overlap accounting, and sentinel-study checks are provided in Supplementary Methods S1 and Supplementary Table S1.

The searches were used to support targeted updating of the evidence base and to verify retrieval of key literature relevant to the review questions; they were not treated as an exhaustive systematic-review screening corpus. Diagnostic and research criteria, neuropathological reference material, mechanistic animal and smaller human studies, disease-specific comparator biomarker studies, pivotal treatment trials, and appropriate-use recommendations were identified through targeted retrieval and reference-list checking. We did not undertake exhaustive title/abstract screening, dual-reviewer study selection, a formal risk-of-bias assessment, or quantitative synthesis. Accordingly, no PRISMA flow diagram or numerical included-study count is reported.

## 3 Post-TBI cognitive trajectories and mechanisms of delayed deterioration

TBI does not produce a single cognitive outcome. Outcomes after mild TBI are heterogeneous: in TRACK-TBI, a minority met a stringent definition of poor cognitive outcome at 1 year, but absence of that endpoint should not be equated with complete recovery (12). Among moderate-to-severe survivors, outcomes range from recovery to progressive deterioration — a heterogeneity that clinical vocabulary currently obscures. Existing frameworks describe TBI outcomes in terms of functional recovery and disability. What we propose here is a complementary way of describing the shape of change over time, intended to organise the question of when a differential diagnostic evaluation is warranted (Table 1; Figure 1). It is a working heuristic, not a validated classification, and no prevalence estimates are offered for any of the patterns described, because none has been measured using a validated classification.

**Table 1:** Proposed patterns of cognitive change after traumatic brain injury.

Trajectory	Clinical pattern	Potential explanatory processes	Distinguishing features	Evidence status
Type I — Apparent Recovery	Apparent recovery within months	Resolution of acute dysfunction and compensatory recovery may contribute; clinical recovery does not exclude residual structural injury	Course consistent with injury severity; no subsequent decline	Descriptive pattern; not a validated category
Type II — Stable Residual Deficit	Partial recovery to an apparently stable plateau	Persistent effects of structural injury and reduced cognitive reserve may contribute	Deficits appear stable on serial assessment and correspond to injury anatomy	Descriptive pattern; not a validated category
Type III — Delayed Deterioration	Recovery or plateau, then new progressive decline months to years later	Potential, unvalidated contributors: tau-related pathology, impaired protein clearance, chronic neuroinflammatory activity, reduced neural reserve, and coexisting age-related neurodegenerative disease. See Section 3.2.	Change in course from stable to progressive, qualitatively distinct from the original deficit	Conceptual; not validated as a distinct biological or etiological subtype
Type IV — Progressive from Onset	No recovery; continuous decline from the acute phase	Severe structural injury, or a pre-existing neurodegenerative process becoming clinically apparent	Continuous rather than biphasic course	Descriptive pattern; competing explanations, including reverse causality, require evaluation

These processes are supported by heterogeneous preclinical, neuropathological, imaging, and epidemiological evidence and have not been validated as a unified mechanism or as defining a distinct clinical subtype.

**Figure 1:**
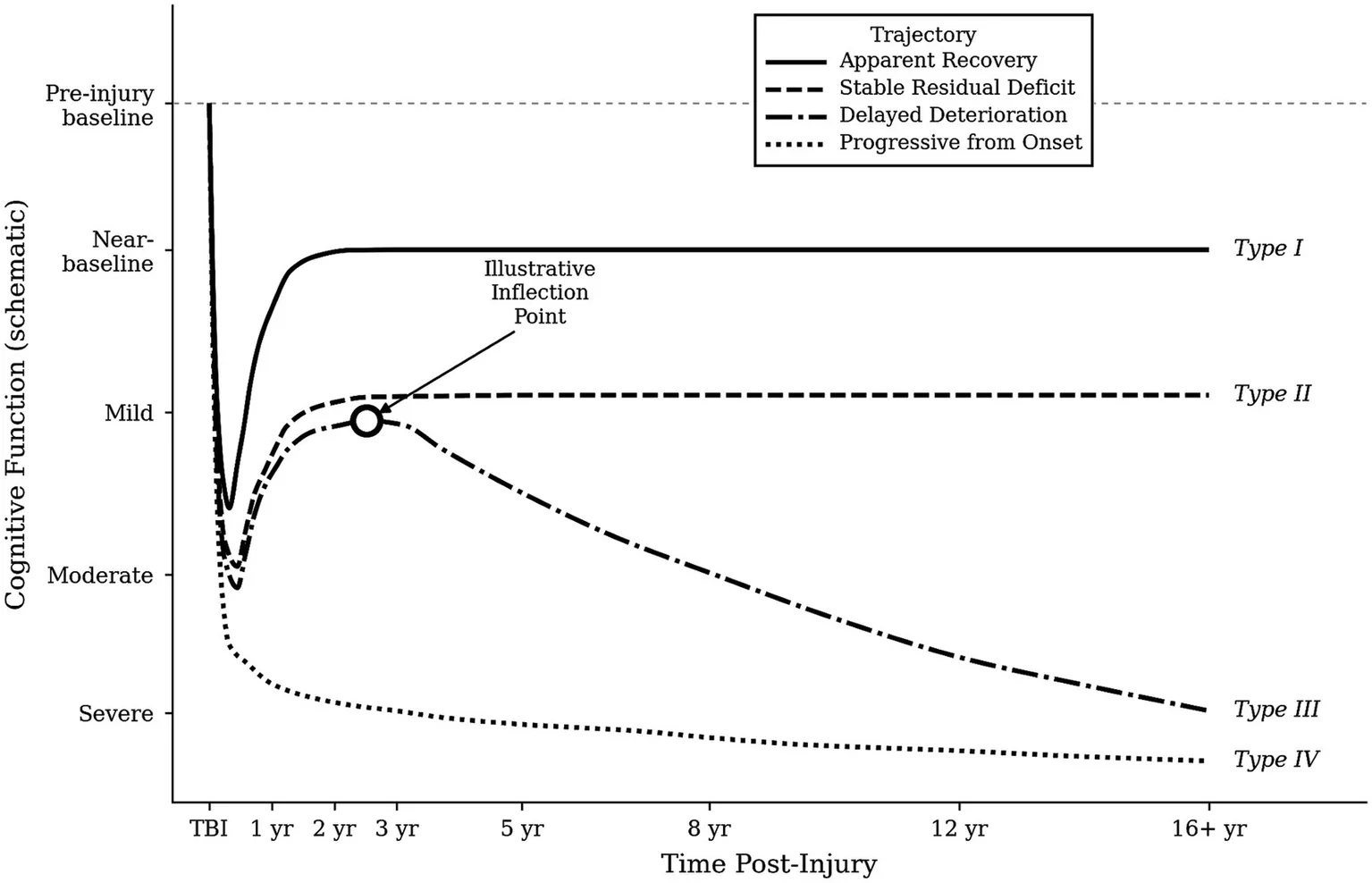
Conceptual schematic of four proposed post-TBI cognitive trajectories. In Type III, the circled illustrative inflection point represents a change from an apparent recovery or stable plateau to new progressive deterioration. This change in course creates the greatest differential diagnostic burden and warrants reassessment for competing post-traumatic, neurodegenerative, and mixed explanations. The trajectories, curve shapes, and timing are schematic and represent unvalidated clinical heuristics rather than empirically established natural histories or fixed diagnostic thresholds. The vertical axis is qualitative, and a documented pre-injury cognitive baseline is often unavailable in practice.

### 3.1 The four trajectories

Type I describes apparent recovery without evidence of subsequent decline; the pattern alone does not indicate a need for intensified neurodegenerative evaluation. Type II describes persistent deficits that appear clinically stable after an initial recovery period, often in the setting of established structural injury. Long-term data are consistent with this being one of several patterns rather than a discrete entity: in a Model Systems cohort followed between 5 and 15 years after injury, the majority showed no change on measures of functional independence, while others improved and others declined, with age and educational attainment rather than initial injury severity emerging as the principal correlates of decline (13). Type IV describes a course progressive from the time of injury, which may reflect pre-existing disease becoming apparent rather than a consequence of the injury. Dementia diagnoses are concentrated in the early period after TBI — in a Swedish nationwide cohort the association was strongest within the first year and attenuated but persisted beyond 30 years — although reverse causality, ascertainment, and pre-existing disease may all contribute (14).

Longitudinal findings do not support a single post-TBI cognitive course. In the ARIC cohort, two or more incident TBIs were associated with faster cognitive decline over 30 years, whereas one incident TBI was not (15). In PROTECT-TBI, lifetime TBI history was associated with domain-specific baseline deficits in later life, particularly in attention and executive function, but not with additional worsening over 4 years (16). Differences in injury number, severity, exposure ascertainment, time since injury, and follow-up duration limit direct comparison. These studies support heterogeneity of long-term cognitive course but do not validate the proposed trajectories or assign an etiology to an individual pattern.

Type III describes delayed progressive deterioration and is the course this review primarily addresses. The patient recovers or reaches a plateau, remains apparently stable for months to years, and then shows progressive decline qualitatively different from what preceded it — the cognitive inflection point illustrated in Figure 1. Caregivers may recognise this change before it is captured by formal testing: a regression of abilities that had returned, a behavioural change, a quality of deterioration distinct from the post-injury plateau. The proportion of survivors following this course has not been established in any prospective cohort using a validated classification. The TRACK-TBI LONG study found that function continued to change between the one-year assessment and follow-up 2 to 7 years after injury, supporting TBI as a chronic evolving condition, but did not isolate delayed deterioration following initial recovery as a distinct subgroup (17).

Patients whose decline emerges years after injury may no longer be receiving structured cognitive follow-up, leaving the later change difficult to reconstruct. A patient who deteriorates 3 or 4 years after injury may return to the clinic for the first time since discharge with no serial cognitive data and no documented record of the plateau maintained before the decline began. The assessment pathway proposed in Section 6 is intended to address that gap by generating a longitudinal record against which later change can be reviewed.

### 3.2 Biological pathways that may link traumatic brain injury to later neurodegeneration

#### 3.2.1 From acute injury to chronic vulnerability

The mechanical forces of a traumatic brain injury produce immediate axonal disruption, blood–brain barrier compromise, and inflammatory activation, and these acute events do not all resolve when clinical stabilisation occurs. Axonal injury in particular may follow a protracted course: in a multicentre cohort of moderate-to-severe TBI, plasma neurofilament light continued to rise after the immediate acute phase and peaked subacutely; it remained elevated in many of the available follow-up samples at 6 and 12 months, and higher subacute concentrations were associated with diffusion-MRI evidence of axonal injury, later functional outcome, and subsequent white-matter atrophy (18). Separate experimental and imaging studies suggest that protein-clearance pathways may be disrupted and inflammatory activity may persist beyond the acute phase. A recent review has considered how these processes might contribute to population-level dementia risk after TBI (6).

Whether these processes act together, sequentially, or independently in an individual remains uncertain. The pathways examined below have been characterised in largely separate literatures that differ in species, injury severity, exposure pattern, and study design. The following subsections therefore distinguish direct findings from mechanistic extrapolation; none has been validated as defining a clinical trajectory in an individual patient.

#### 3.2.2 Tau-related pathology

The most direct experimental evidence comes from a mouse model. Inoculation of naive mice with brain homogenate from injured animals produced tau pathology, synaptic loss, and memory impairment, consistent with experimental transmission of tau pathology in that model (19).

The corresponding human data are more limited and are of a different kind. In an autopsy series of long-term survivors of a single moderate-to-severe TBI, the proportion of cases with any tau pathology did not differ from controls; what differed was its extent and anatomical distribution, with widespread pathology in 9 of 15 injured cases (19). Whether tau pathology propagates from cell to cell in the injured human brain has not been demonstrated, and the authors of that work identify human confirmation as an outstanding question.

Evidence derived from repetitive head impacts requires separate handling. In a retrospective autopsy cohort of 750 individuals comprising 300 former athletes and 450 non-athletes, chronic traumatic encephalopathy or CTE-like features were identified in 42 cases and were associated with contact-sport participation; a documented clinical diagnosis of head trauma was not itself more strongly associated with CTE pathology, and the authors interpreted their findings as more consistent with repetitive exposure than with a single later head injury (20). This evidence therefore supports an association with repetitive head-impact exposure and should not be extrapolated to the consequences of a single TBI.

#### 3.2.3 Impaired protein clearance

In a mouse closed-skull model of moderate TBI, glymphatic function fell by approximately 60% and remained impaired for at least 1 month after injury; deletion of aquaporin-4 worsened the clearance deficit and was accompanied by greater tau accumulation and neurodegeneration (21).

Two limits follow directly. First, the longest interval examined in that model was approximately 1 month, so the evidence does not speak to persistence over years. Second, no human study has established the duration of post-traumatic glymphatic impairment or its clinical consequences. The relevance of post-traumatic sleep disturbance to glymphatic function and protein accumulation in humans remains untested.

#### 3.2.4 Chronic neuroinflammatory activity

Increased binding of the translocator protein ligand [11C]PK11195 has been demonstrated in a small cross-sectional positron emission tomography study of 10 patients with moderate-to-severe TBI, each imaged once at an interval ranging from approximately 1 to 17 years after injury, with elevated binding in the thalamus, putamen, occipital cortex, and internal capsule, and with thalamic binding related to performance on some cognitive measures (22).

The design constrains what can be concluded. Because each patient contributed a single scan, the study demonstrates that elevated binding can be observed up to 17 years after injury in selected patients; it does not establish that the signal persists continuously within an individual. An increased TSPO-ligand signal does not identify a specific microglial phenotype and therefore cannot establish microglial “priming.” Whether this activity amplifies tau propagation, sustains barrier dysfunction, or accounts for clinical deterioration after a subsequent minor injury has not been examined in these data.

Inflammation and white matter degeneration can persist for years after a single TBI, as demonstrated in post-mortem tissue by Johnson et al. (23).

#### 3.2.5 Human evidence and the limits of causal attribution

In 532 consecutive autopsies from a community-based cohort of adults aged 65 years or older who were free of dementia at enrolment, only three cases (0.6%) showed neuropathological features of CTE, each confined to a single temporal or parietal region (24). None of the three had a recorded history of TBI with loss of consciousness before developing dementia. Injuries without loss of consciousness and repetitive head-impact exposure were, however, incompletely ascertained.

Two studies from the National Alzheimer’s Coordinating Center distinguish earlier clinical expression from subsequent progression. In an autopsy-confirmed sample of 2,133 individuals with Alzheimer disease, a remote history of TBI with loss of consciousness was associated with symptom onset 2.34 years earlier and diagnosis 2.83 years earlier (25). In a separate cohort of 2,719 individuals with mild cognitive impairment followed for up to 8 years, TBI history was not associated with progression to Alzheimer disease or with the rate of decline among those who progressed, although mild cognitive impairment was diagnosed 2.6 years earlier in the TBI group (26). These observations suggest that earlier clinical presentation does not necessarily imply faster subsequent progression.

They do not determine whether TBI triggered, accelerated, revealed, or was unrelated to the underlying disease process in an individual patient. The pathways described in this section are therefore hypothesis-generating accounts of biological plausibility rather than demonstrated causal mechanisms in an individual case.

## 4 The differential diagnostic challenge

### 4.1 Why existing criteria fall short

When delayed progressive decline appears in a patient with a TBI history, the available diagnostic frameworks were not written to answer the question being asked. The 2024 Alzheimer’s Association revised criteria define Alzheimer disease biologically and permit diagnosis on the basis of an abnormal validated Core 1 biomarker; the accompanying staging scheme explicitly accommodates copathology and reserve as modifiers of the relationship between biological and clinical stage (27). These criteria identify Alzheimer pathology. They do not determine the relative contribution of an earlier injury, or of other coexisting processes, to the clinical syndrome, and their authors state that they are not intended as clinical practice guidelines. DSM-5-TR Major Neurocognitive Disorder Due to TBI requires that impairment be attributable to the injury, without guidance on establishing attribution years later (28). The 2021 NINDS research criteria for traumatic encephalopathy syndrome (TES) require substantial exposure to repetitive head impacts, progressive core clinical features, and that those features are not fully accounted for by another neurologic, psychiatric, or medical condition; a coexisting neurodegenerative diagnosis does not automatically exclude the syndrome, although the criteria are not met when another disorder fully accounts for the core features (29). Those criteria were developed to facilitate research into chronic traumatic encephalopathy, were informed by clinicopathological data from neuropathologically diagnosed cases, and require validation in living populations.

The practical difficulty is therefore not that the frameworks contradict one another, but that each identifies a different aspect of what may be a mixed clinical presentation, and none was constructed to apportion contribution between them. A patient with a moderate TBI history, progressive decline beginning 3 years after injury, and an abnormal Core 1 biomarker can be characterised biologically under the Alzheimer criteria. What remains unresolved is the contribution of the injury.

Population-level associations vary according to injury severity and exposure pattern. A single mild TBI followed by apparent recovery should not be assigned the same evidentiary weight as moderate-to-severe injury or substantial repetitive head-impact exposure in contact-sport, military, or other settings. These features may inform the level of clinical suspicion and the breadth of the differential evaluation, but they do not provide a validated individual prior probability or establish causation, and the features in Table 2 should be read in that light.

**Table 2:** Illustrative features across the clinical situations under consideration.

Feature	Stable post-traumatic course	Delayed progressive decline, cause unresolved	Alzheimer disease	Repetitive head-impact exposure with suspected TES
Temporal pattern	Deficits present after injury; no subsequent progression on serial assessment	Recovery or plateau, then progressive change after an interval	Progressive onset consistent with Alzheimer disease; its temporal relation to an injury does not determine the injury’s contribution	Progressive features after substantial repetitive exposure
Cognitive profile	Generally maps to injury anatomy	Change extending beyond the pattern attributable to the injury	Often amnestic, although non-amnestic presentations occur; interpreted through established Alzheimer disease diagnostic pathways	Cognitive impairment in episodic memory or executive function, and/or neurobehavioural dysregulation (29)
Course	Non-progressive on available follow-up	Progressive	Progressive, with substantial individual variability	Progressive
Neuroimaging	Encephalomalacia at the injury site without progressive atrophy elsewhere	Atrophy extending beyond the injury anatomy raises the possibility of an additional process	Pattern consistent with Alzheimer disease	No imaging finding currently identifies CTE during life (45)
Principal caution	—	No biomarker profile identifies an injury-driven process	Diagnosis follows the established pathway; see Section 5	Requires that features are not fully accounted for by another condition; CTE cannot be confirmed during life

Columns describe clinical situations under consideration, not validated etiological categories. Features are typical rather than defining; none is specific, and no single feature or combination establishes an etiology in an individual patient. Post-TBI NfL and GFAP kinetics vary with injury severity, assay, and sampling window and have not been validated to distinguish these situations. Alzheimer disease biomarkers may establish biological Alzheimer pathology but do not determine the contribution of a prior injury. See Sections 4.2 and 5.

Contemporary TBI classification is also moving beyond reliance on Glasgow Coma Scale–based labels alone. The 2025 NIH–NINDS TBI Classification and Nomenclature Initiative proposed the CBI-M framework for multidimensional characterization during the acute phase, integrating clinical findings, blood-based biomarkers, imaging features, and patient-level modifiers (30). This framework may improve reconstruction of the nature and context of the original injury when such information was collected. It was not designed to classify delayed cognitive trajectories or to determine whether TBI caused or contributed to neurodegenerative disease years later. Acute injury characterization and later etiological assessment therefore remain related but distinct tasks.

### 4.2 Clinical and biomarker differentiation

The clinical differentiation in this section begins with temporal course and cognitive phenotype; coexisting vascular disease is considered separately, including assessment of potentially modifiable vascular risk factors. Biomarker and imaging evidence is examined in Section 5 and summarized alongside these features in Table 2. Temporal pattern provides the first orientation: onset within the first year after injury more often reflects direct injury effects or pre-existing pathology becoming apparent. Decline emerging after a period of recovery carries the greatest diagnostic uncertainty and is the situation this review primarily addresses.

The cognitive profile is a second orientation point. Post-traumatic deficits generally map to injury anatomy. Alzheimer disease commonly presents with an amnestic syndrome, although non-amnestic phenotypes occur; disease-specific biomarkers and imaging should be interpreted through established Alzheimer disease diagnostic pathways. Where a patient with frontal injury presents with predominantly posterior cortical deficits, the mismatch between injury anatomy and symptom pattern is a reason to evaluate further. None of these patterns is specific, and none identifies an etiology on its own.

Vascular injury and coexisting cerebrovascular disease should be assessed as potential contributors to delayed cognitive decline rather than being subsumed under the categories in Table 2. Susceptibility-weighted imaging may identify traumatic microhaemorrhagic burden, while conventional vascular imaging findings and vascular risk factors may indicate an additional pathway requiring independent evaluation. Coexisting vascular risk factors are potentially modifiable and warrant independent assessment, even when their contribution to the observed cognitive course cannot be quantified.

## 5 Biomarker interpretation in the post-TBI context

Blood biomarkers measured after TBI serve different purposes according to the sampling interval and clinical question. Acute panels including GFAP, UCH-L1, NfL, and total tau have been evaluated for characterising acute intracranial injury; in a TRACK-TBI Pilot analysis of plasma collected within 24 h, these markers showed varying ability to distinguish abnormal from normal head CT findings (31). That study evaluated acute assay performance and CT abnormalities rather than longitudinal kinetics, cognitive decline, or etiological attribution. Acute injury characterisation and the later assessment of coexisting or superimposed neurodegenerative disease are therefore different tasks.

No single biomarker measurement or serial pattern has been validated to assign a post-TBI cognitive trajectory or determine the cause of decline in an individual patient. Interpretation depends on the clinical course, injury severity, sampling interval, assay characteristics, and the use of validated disease-specific diagnostic pathways. The interpretive roles and limitations of selected blood biomarkers across assessment intervals are summarised in Table 3. This section therefore examines what fluid biomarkers and neuroimaging can and cannot contribute to the evaluation of competing explanations, rather than advocating routine serial biomarker monitoring.

**Table 3:** Interpretive roles and limitations of selected blood biomarkers after TBI.

Biomarker	Acute and subacute behaviour	Longer-term behaviour	Biological interpretation	Potential value	Major limitation	Refs
GFAP	Peaks within the first day after injury (median peak 12590.5 pg./mL) and falls after the subacute period	Among participants with samples available at the respective follow-up visit, plasma GFAP exceeded the healthy-control mean by more than 2 SD in 18 of 53 at 6 months and 8 of 25 at 12 months. These denominators represent the available follow-up samples, not the full cohort	An astroglial protein. In the cerebral-microdialysis subgroup, plasma GFAP did not correlate with GFAP in brain extracellular fluid. The authors hypothesised that plasma concentrations might partly reflect release associated with blood–brain barrier injury, but this mechanism remains unconfirmed	Contributes to acute injury characterisation; a weaker but significant predictor of functional outcome at 6 and 12 months	Persistent elevation has not been validated as a marker of a post-traumatic neurodegenerative process. Age contributed to concentrations at 6 months, and no validated within-person change threshold permits an individual result to indicate progressive disease	18
NfL	Rises after injury and peaks subacutely, between 10 days and 6 weeks and at around 20 days in this cohort (median peak 507.3 pg./mL), in contrast to tau, UCH-L1, S100B and GFAP, which peaked within the first day	Among participants with samples available at the respective follow-up visit, plasma NfL exceeded the healthy-control mean by more than 2 SD in 45 of 53 at 6 months and 13 of 25 at 12 months. These denominators represent the available follow-up samples, not the full cohort	An axonal cytoskeletal protein. Plasma concentrations correlated with concentrations in brain extracellular fluid and with diffusion-MRI measures of axonal injury, and an aligned experimental model showed a dose–response relationship to histopathologically defined axonal injury. Elevation is not specific to trauma	The subacute peak was the strongest single predictor of functional outcome at 6 and 12 months and the only blood marker that predicted white-matter atrophy over the 6-to-12-month interval	Persistent elevation at 6 or 12 months cannot by itself identify delayed neurodegeneration or establish an individual longitudinal trajectory. No validated within-person change threshold exists, and the study did not test NfL against a clinical diagnosis of post-traumatic neurodegenerative disease	18
p-tau181	Acute changes after TBI are incompletely characterised. Transient early elevation has been reported in mild TBI and sports-related concussion, but findings are limited and assay- and cohort-dependent	Persistent or delayed elevation may support evaluation for coexisting Alzheimer-type pathology, but post-TBI longitudinal reference trajectories are not established	An Alzheimer-associated tau biomarker when interpreted within a validated Alzheimer diagnostic pathway; it is not specific for the contribution of a previous TBI	May contribute to evaluation for coexisting biological Alzheimer disease when interpreted with clinical context and other validated biomarkers	Neither the concentration nor its timing identifies injury-driven neurodegeneration or establishes when Alzheimer pathology began	33, 34, 27

Study-population note: Graham et al. (18) studied adults with a single moderate-to-severe TBI; patients with pre-existing neurological disease or a previous TBI requiring hospitalisation were excluded. Six- and 12-month blood sampling was performed only at a subset of sites. The chronic biomarker findings therefore should not be generalised to recurrent TBI or pre-existing neurodegenerative disease.

### 5.1 Phosphorylated tau biomarkers in post-TBI evaluation

Plasma p-tau181 findings after TBI vary with injury severity, sampling interval, assay, and cohort. Transient early elevation has been reported after mild TBI and sports-related concussion, whereas findings from moderate-to-severe TBI do not support a uniform first-year response. These observations do not establish a severity-independent post-TBI trajectory (32, 33).

Persistent or delayed elevation may support evaluation for coexisting biological Alzheimer disease, but only within a validated Alzheimer diagnostic pathway and in conjunction with clinical context and other validated biomarkers (27). Within that framework, accurate plasma p-tau217 assays receive particular emphasis among Core 1 biomarkers; however, the post-TBI evidence reviewed here does not establish a TBI-specific discriminatory role for p-tau217. Plasma p-tau181 is not specific for the contribution of a previous TBI, and an isolated concentration or its timing cannot establish when Alzheimer pathology began or whether it explains the observed cognitive course. Neither a blood p-tau result nor an Alzheimer-type tau-PET distribution identifies CTE pathology during life.

### 5.2 Neurofilament light and GFAP

Longitudinal kinetics after moderate-to-severe TBI have been characterised in the BIO-AX-TBI cohort, although their generalisability across injury severities remains uncertain.

Plasma neurofilament light does not behave as a purely acute marker. In that cohort, concentrations continued to rise after the immediate acute period and peaked subacutely, in contrast to total tau, UCH-L1, S100B and GFAP, which peaked within the first day; the authors suggest that sampling 2 to 4 weeks after injury may be the most informative window for assessing axonal injury (18). Concentrations remained elevated in many of the available follow-up samples at 6 and 12 months. Higher subacute concentrations were associated with diffusion-MRI measures of axonal injury, with white-matter atrophy over the subsequent 6 to 12 months, and with functional outcome at 1 year.

These kinetics constrain interpretation. Because persistent elevation was common in that cohort, failure of neurofilament light to normalise cannot by itself identify a delayed neurodegenerative process; that study was not designed to evaluate the marker against such an outcome. In a prospective mixed-mechanism mild-TBI cohort, plasma neurofilament light remained elevated at 3 months but returned to control levels by 12 months; this trajectory should not be generalised across injury severity (34). Serial interpretation is more defensible when prior measurements from the same patient are available, although no validated within-person change threshold currently distinguishes residual injury from a superimposed new process. Neurofilament light is therefore best interpreted as a measure of neuroaxonal injury burden that is sensitive but not etiologically specific.

GFAP shows a different pattern. It peaks within the first day after injury, but in the same moderate-to-severe cohort plasma GFAP remained raised at 12 months in a minority of the samples available at that visit (18). In the 18-patient acute cerebral-microdialysis subgroup, plasma GFAP did not correlate with GFAP in brain extracellular fluid. The authors hypothesised that plasma concentrations might partly reflect release associated with blood–brain barrier injury, but this mechanism remains unconfirmed. Persistent GFAP elevation should therefore not be interpreted as evidence of a specific post-traumatic neurodegenerative process.

### 5.3 Structural and molecular neuroimaging

Structural and molecular imaging provide complementary information alongside plasma biomarkers. Stable encephalomalacia at the injury site with no progressive atrophy elsewhere is consistent with a static deficit. Progressive hippocampal or cortical atrophy beyond the injury anatomy raises the possibility of an additional neurodegenerative process, and posterior cortical thinning in a patient with predominantly frontal injury raises the same question. Where tau-PET is available it can demonstrate an Alzheimer-type distribution consistent with Braak topography. Updated appropriate-use criteria judged tau PET appropriate in 5 of 17 clinical scenarios; more were judged uncertain or rarely appropriate for tau PET, with six scenarios in each category (35). Tau-PET should not be used to identify CTE pathology in life (see Section 5.1).

## 6 Clinical framework: monitoring, differential diagnosis, and treatment

This section considers post-TBI cognitive monitoring and differential diagnosis in three parts: a proposed longitudinal assessment pathway, prognostic and management implications, and the medicolegal dimension.

### 6.1 A proposed longitudinal assessment pathway

Serial assessment can describe change over time more reliably than a single assessment, but the pathway proposed here does not assume that an observed course maps to a validated trajectory. It is built primarily on clinical, cognitive, and functional follow-up, with laboratory and imaging investigations added according to clinical indication. It has not been prospectively evaluated and is offered as a testable framework rather than as a standard of care.

After the acute injury, injury severity and imaging findings are documented as they would be in routine trauma care. Blood biomarkers obtained during acute management, where used, should be interpreted primarily as measures of acute injury and not as a validated baseline for later etiologic attribution. At a post-acute review, generally within the first few months, a brief cognitive screen together with a structured account of functional change provides a practical reference for subsequent comparison. A more comprehensive neuropsychological assessment at 6 to 12 months may serve as the principal reassessment point for determining whether deficits appear stable or evolving. Where performance appears stable, rehabilitation and usual clinical follow-up can continue as indicated. Where new or progressive decline is identified, evaluation should follow established diagnostic pathways for the competing condition under consideration rather than a fixed post-TBI biomarker panel. The illustrative reassessment windows in this proposed pathway are summarised in Table 4.

**Table 4:** Illustrative reassessment windows in the proposed assessment pathway.

Illustrative window	Focus	Clinical and cognitive assessment (primary)	Investigations, if clinically indicated (adjunct)
Acute	Injury characterisation	Injury severity, level of consciousness, post-traumatic amnesia, neurological examination	Imaging as directed by routine trauma care. Blood biomarkers, where used, measure acute injury; see Section 5
Post-acute (first few months)	Establishing a reference	Brief cognitive screen; structured account of functional change; activities of daily living	Investigations as clinically indicated; no scheduled biomarker panel
6–12 months	Principal reassessment	Comprehensive neuropsychological assessment compared with the post-acute reference; determine whether deficits appear stable or evolving	Where decline is identified, investigations follow established diagnostic pathways for the condition under consideration
Subsequent	Continued follow-up	Clinical and cognitive review at intervals determined by the clinical course	As clinically indicated. Repeat NfL or GFAP measurement is not proposed as routine surveillance

The intervals and assessment components shown are illustrative rather than prescriptive and have not been prospectively validated. No biomarker or imaging finding in this table establishes a trajectory or an etiology. See Sections 5.2 and 6.1.

Current evidence does not support routine repeat measurement of NfL or GFAP solely to assign a trajectory or etiology. Their kinetics vary with injury severity and sampling interval, and no validated reference trajectory or within-person change threshold permits an individual result to be used for etiologic classification. Where these biomarkers are obtained for a clinical indication, they should be interpreted in the context of injury severity, time since injury, and any available prior measurements. APOE genotyping, when relevant, may inform ARIA risk assessment before anti-amyloid therapy but should not be used to adjudicate whether cognitive decline was caused by TBI.

The cognitive inflection point deserves particular attention because it may first become apparent in the clinical history before it is captured by formal testing. A caregiver may describe abilities that had returned but are now receding, or a qualitative change in function from the preceding plateau. Such reports may precede measurable change and may arise despite stable structural imaging. This discrepancy does not itself establish progression, but it should prompt comparison with prior function and evaluation of competing explanations. Dismissing a progressive process may delay appropriate assessment, whereas over-interpreting normal variation may generate unnecessary investigation and illness attribution. Systematic documentation of patient- and caregiver-reported functional change alongside formal cognitive testing can support earlier recognition and create a longitudinal record against which subsequent change can be assessed.

### 6.2 Prognostic and management implications

Management implications differ according to the condition identified, and the honest starting point is that current evidence supports fewer distinctions than the categories might suggest (Table 5). Where deficits appear stable on serial assessment, the clinical priority is rehabilitation and symptom-directed care. Where decline is new or progressive, prognosis follows the condition eventually identified rather than any trajectory label; assigning a distinct prognosis to a category that has not been validated would not be justified. Observational evidence does suggest that a history of TBI is associated with earlier clinical onset of Alzheimer disease in autopsy-confirmed cohorts (25) and with an increased risk of dementia (36). Family counselling should convey what these findings do and do not mean: an association with earlier onset at the group level is not a prediction for an individual, and it does not establish that the injury caused the disease.

**Table 5:** Management implications according to the established or suspected clinical condition.

Clinical situation	Prognostic interpretation	Current management	Disease-specific considerations
Stable post-traumatic deficits	Course appears stable on serial clinical assessment; long-term individual change remains possible (7)	Rehabilitation and symptom-directed care	Biomarkers are not required to assign a trajectory
New or progressive decline after TBI, cause unresolved	Requires evaluation of competing and potentially mixed causes	Reassess reversible contributors; neuropsychological and disease-specific evaluation	No blood biomarker profile identifies post-traumatic neurodegeneration
Established biological or clinical Alzheimer disease in a patient with TBI history	Prognosis and treatment follow the established Alzheimer disease pathway	Alzheimer-directed symptomatic and disease-specific management as appropriate	APOE may inform ARIA safety assessment, not causal attribution (41)
Repetitive-head-impact exposure with suspected traumatic encephalopathy syndrome	CTE cannot be confirmed during life; competing diagnoses must be evaluated (29, 45)	Symptom-directed and supportive care	Do not extrapolate evidence from repetitive-impact cohorts to a single TBI

Rows describe clinical situations, not validated etiological categories. Prognostic statements apply at group level and are not individual predictions. No entry in this table establishes that later cognitive decline was caused by an earlier injury.

There is no approved disease-modifying therapy for post-traumatic neurodegeneration. Two tau-directed monoclonal antibodies did not demonstrate clinical benefit in phase 2 trials conducted in other tauopathies: gosuranemab in progressive supranuclear palsy (37) and semorinemab in prodromal-to-mild Alzheimer disease (38). Neither trial evaluated post-traumatic neurodegeneration, and these findings should not be interpreted as establishing inefficacy in that population. Strategies targeting neuroinflammation show preclinical promise, but none has demonstrated clinical efficacy after TBI in humans. Despite this therapeutic limitation, the diagnostic distinction remains clinically relevant.

One implication is that diagnosis helps determine which treatments should not be given. Anti-amyloid therapies have been studied and authorised for early symptomatic Alzheimer disease with confirmed amyloid pathology (39, 40). Where such treatment is available, eligibility and safety assessment should follow the relevant regulatory label and agent-specific guidance. A patient whose clinical syndrome and amyloid status have not been shown to meet those criteria should not be assumed to be eligible for treatment. Because these agents carry a recognised risk of amyloid-related imaging abnormalities, treatment outside the population in which benefit and safety were established would expose a patient to risk without a corresponding evidence base for benefit. APOE genotype, concomitant medications, and baseline MRI findings — including the burden of cerebral microhaemorrhages, superficial siderosis, and other haemorrhagic lesions — inform treatment eligibility and ARIA-related safety assessment (41); none of these is a means of deciding whether an injury caused the decline.

A second implication concerns the design of future studies. The pivotal anti-amyloid trials did not establish efficacy or safety specifically in patients being evaluated for post-traumatic cognitive decline, and trial- or guidance-based MRI exclusions may limit direct extrapolation to some patients with structural sequelae of TBI. Trials directed at post-traumatic neurodegeneration will require prospectively defined clinical phenotypes and independently validated biomarker or imaging endpoints; the trajectories proposed here and current blood-biomarker patterns are not yet suitable as stand-alone eligibility criteria. Describing patients consistently, with documented baselines and serial assessments, is what would make such trials feasible.

Current management remains supportive and risk-directed rather than disease-modifying. Management of vascular risk factors, attention to sleep disturbance, and measures to prevent further head injury are reasonable components of care after TBI on established clinical grounds, independent of any specific mechanistic rationale (3). Rehabilitation should be individualised according to current deficits, functional goals, and the condition identified (42); the evidence does not define a trajectory-specific window in which it is most effective. Cholinesterase inhibitors are considered within the established pathway for Alzheimer disease and are not supported for post-traumatic cognitive impairment in the absence of that diagnosis. The lack of a specific pharmacological treatment does not remove the value of an accurate diagnosis; it defines what still needs to be studied.

### 6.3 The medicolegal dimension

Questions of attribution also arise in medicolegal settings, where clinicians may be asked whether current cognitive impairment represents a sequela of the documented injury, an independent disease process, or a combination of both. Industrial injury claims, road traffic compensation, and disputes over the duration of covered treatment may turn on some version of this question. A patient whose structural imaging is unchanged but whose function is declining presents the difficulty in its clearest form. Stable imaging does not exclude a progressive process, and it does not by itself indicate one; the question cannot be resolved from a single modality.

A defensible assessment begins with the documented pre-injury functional and cognitive baseline, followed by injury severity and characteristics, the temporal sequence of recovery and later change, serial clinical and neuropsychological findings, structural imaging, and evaluation of competing explanations. Contemporaneous documentation of patient- and caregiver-reported change is valuable because it makes the timing and nature of an apparent inflection point reviewable rather than dependent on retrospective recollection. Records created during routine follow-up, before a question of attribution has arisen, are generally less susceptible to recall and attribution bias than later retrospective reconstructions.

Biomarker and imaging findings may strengthen or weaken particular competing diagnoses, but they do not independently establish that later cognitive decline was caused by the earlier injury. Biomarkers may support evaluation for an established coexisting disease pathway, such as biological Alzheimer disease; absence of such evidence does not constitute positive evidence of injury-driven neurodegeneration. No currently validated neurofilament light, GFAP, phosphorylated-tau, imaging, or APOE profile positively identifies post-traumatic neurodegeneration in an individual patient. APOE genotype may inform ARIA risk assessment before anti-amyloid treatment, but it should not be used as a causal adjudicator.

In older patients with fall-related TBI, reverse causality must be considered: impaired gait, attention, judgement, or cognition preceding the injury may have contributed to the fall. Considerations that raise this possibility include an unwitnessed or unexplained ground-level fall, particularly where preceding functional or cognitive change is documented or suspected; the absence of a documented pre-injury baseline; a course that was progressive from the time of injury rather than showing recovery followed by later change; and biomarker evidence supporting coexisting Alzheimer pathology within the first months after injury. Such biomarker evidence may strengthen that competing diagnosis but does not establish when the pathology began. Conversely, a documented pre-injury baseline and a subsequent change temporally related to the injury may support a contributory relationship, but they do not by themselves distinguish direct causation, acceleration of an existing disease, reduced reserve, or mixed pathology. The distinction between an outcome caused by the injury and one to which the injury contributed is central and is often the point on which an opinion turns.

A medicolegal opinion should distinguish among direct injury sequelae, possible contribution or acceleration, an independent neurodegenerative disorder, mixed causation, and an indeterminate relationship. The opinion should identify which findings are documented, which are inferential, which competing explanations remain, and how the absence of pre-injury data limits confidence. Where these possibilities cannot be reliably separated, stating that uncertainty explicitly is more scientifically defensible than forcing a binary attribution.

## 7 Limitations, future directions, and conclusion

### 7.1 Limitations

As a narrative review, the literature synthesis is subject to the inherent limitations of the method: study selection reflects author judgment rather than a pre-registered protocol, quantitative pooling was not undertaken because the included literature spans heterogeneous populations, exposure patterns, outcomes, and study designs, and publication bias cannot be formally assessed. The structure and reporting of this review were informed by the domains of the SANRA quality-assessment scale, and it should be interpreted as a structured expert synthesis rather than a systematic review. The evidence base draws predominantly from North American and European cohorts. Meta-analytic subgroup data suggest that the association between TBI and dementia may differ by population — a higher pooled estimate was reported among studies of Asian populations, though between-study heterogeneity was very high — and the generalizability of biomarker thresholds to Asian populations, where TBI epidemiology, APOE allele frequencies, and healthcare pathways differ, has not been established and warrants dedicated study.

The epidemiological foundation for attributing Alzheimer disease to an individual TBI remains uncertain, particularly after a single mild injury. As noted in the Introduction, population-level evidence does not by itself establish causation in an individual patient. The literature also contains several relevant null findings. In subgroup analysis by Gu et al. (2), TBI with loss of consciousness was not associated with dementia risk (pooled OR 0.96, 95% CI 0.84–1.09). In the 3C Dijon cohort, self-reported TBI with loss of consciousness was not associated with incident dementia over follow-up (HR 0.90, 95% CI 0.60–1.36) (43). In a nationwide longitudinal cohort, TBI history and characteristics were not associated with subsequent neurocognitive trajectories or conversion to mild cognitive impairment or dementia (44). These findings do not establish absence of risk, because the studies differ in exposure ascertainment, populations, outcomes, and susceptibility to selection and misclassification, and heterogeneity across the pooled studies was substantial. They do argue against treating a history of loss of consciousness or of TBI alone as sufficient evidence of individual-level causation. Schaffert et al. (25), by contrast, examined individuals already selected for autopsy-confirmed Alzheimer disease and reported earlier clinical onset among those with a history of TBI with loss of consciousness, addressing clinical expression rather than incident risk or subsequent neurocognitive trajectory. Together, these findings support calibrated rather than uniform prior probability: a single mild TBI in a younger patient warrants different suspicion from moderate-to-severe or repetitive injury.

Across several of the epidemiological and neuropathological studies reviewed here, exposure was defined primarily as a recorded or self-reported TBI with loss of consciousness. Injuries without loss of consciousness and repetitive subconcussive exposure may therefore have been classified as unexposed or incompletely ascertained, so null findings apply to those operational exposure definitions rather than to all previous head-impact exposure.

The biomarker limitations extend beyond reference ranges. Thresholds described for the chronic post-TBI context have not been validated against post-mortem neuropathological confirmation in post-TBI cohorts, and reference ranges established in sporadic Alzheimer disease populations may not transfer. Beyond this, the kinetics of neurofilament light and GFAP after TBI vary with injury severity, sampling window, and assay, and no validated reference trajectory or within-person change threshold currently permits an individual result to be used for etiologic classification. The differential interpretation discussed in this review should therefore be read as a framework for structuring evaluation, not as a means of assigning etiology in an individual patient.

The mechanistic evidence is heterogeneous in a way that limits what can be concluded from it. Tau propagation, impaired protein clearance, and chronic neuroinflammatory activity have been demonstrated in different populations and predominantly in animal, neuropathological, or small cross-sectional studies. They have not been shown to form a unified causal cascade, and evidence derived from repetitive head-impact exposure should not be extrapolated to single TBI.

The four-trajectory framework proposed here has not been prospectively validated. It is a conceptual clinical heuristic derived from the reviewed literature, not an empirically derived taxonomy with established prevalence, sensitivity, specificity, or prognostic performance, and no prevalence estimates are offered for that reason. TRACK-TBI LONG documented continuing functional change between the one-year assessment and follow-up conducted 2 to 7 years after injury (17), but it did not isolate the specific sequence of initial recovery or plateau followed by delayed progressive deterioration. The framework should therefore be applied as a hypothesis-generating structure for longitudinal assessment, not as a validated diagnostic guideline.

CTE cannot currently be confirmed in living patients (45).

The principal evidence gaps are also reflected in national research priorities (46), which call for standardized characterization of lifetime head-impact exposure, harmonized longitudinal and multimodal phenotyping, validation of biomarkers across diverse exposure histories, and integration of antemortem clinical data with postmortem neuropathology. These priorities underscore that the proposed trajectories and assessment pathway remain hypotheses for prospective evaluation rather than established diagnostic instruments.

### 7.2 Conclusion

Post-TBI cognitive decline should be approached as a longitudinal diagnostic problem rather than attributed to the prior injury by default. What has improved is not the ability to assign a cause, but the ability to structure the question: to establish a functional reference early, to document change as it is reported rather than reconstructing it later, to recognise clinically meaningful change from the preceding course, and to evaluate competing explanations.

Blood biomarkers and imaging contribute to that structure without resolving it. They can support or weaken the case for a specific coexisting disease, most usefully for biological Alzheimer disease, where identification carries direct consequences for eligibility and safety in treatment decisions. They cannot, at present, identify injury-driven neurodegeneration in an individual patient, and no combination of neurofilament light, GFAP, phosphorylated tau, imaging pattern, or APOE genotype should be read as doing so.

What follows from an accurate assessment is therefore more modest than a treatment algorithm, and still worth having. Where Alzheimer disease is established, management follows the corresponding disease-specific pathway. Regardless of whether Alzheimer disease is identified, vascular risk factor management, attention to sleep, prevention of further head injury, and individualised rehabilitation remain available on ordinary clinical grounds. Where the cause remains unresolved, saying so — to the patient, to the family, and where relevant in a medicolegal opinion — is not a failure of assessment but an accurate description of what is currently knowable.

## References

[1] Gardner RC Bahorik A Kornblith ES Allen IE Plassman BL Yaffe K. Systematic review, meta-analysis, and population attributable risk of dementia associated with traumatic brain injury in civilians and veterans. J Neurotrauma. (2023) 40:620–34. doi: 10.1089/neu.2022.0041, 36305374 PMC10325813

[2] Gu D Ou S Liu G. Traumatic brain injury and risk of dementia and Alzheimer's disease: a systematic review and meta-analysis. Neuroepidemiology. (2022) 56:4–16. doi: 10.1159/000520966, 34818648

[3] Livingston G Huntley J Liu KY Costafreda SG Selbæk G Alladi S . Dementia prevention, intervention, and care: 2024 report of the lancet standing commission. Lancet. (2024) 404:572–628. doi: 10.1016/S0140-6736(24)01296-0, 39096926

[4] Graham A Livingston G Purnell L Huntley J. Mild traumatic brain injuries and future risk of developing Alzheimer's disease: systematic review and meta-analysis. J Alzheimer's Dis. (2022) 87:969–79. doi: 10.3233/JAD-220069, 35491789

[5] Weiner MW Harvey D Landau SM Veitch DP Neylan TC Grafman J . Traumatic brain injury and post-traumatic stress disorder are not associated with Alzheimer's disease pathology measured with biomarkers. Alzheimers Dement. (2023) 19:884–95. doi: 10.1002/alz.12712, 35768339 PMC10269599

[6] Graham NSN Sharp DJ. Understanding neurodegeneration after traumatic brain injury: from mechanisms to clinical trials in dementia. J Neurol Neurosurg Psychiatry. (2019) 90:1221–33. doi: 10.1136/jnnp-2017-317557, 31542723 PMC6860906

[7] Wilson L Stewart W Dams-O'Connor K Diaz-Arrastia R Horton L Menon DK . The chronic and evolving neurological consequences of traumatic brain injury. Lancet Neurol. (2017) 16:813–25. doi: 10.1016/S1474-4422(17)30279-X, 28920887 PMC9336016

[8] Brett BL Gardner RC Godbout J Dams-O'Connor K Keene CD. Traumatic brain injury and risk of neurodegenerative disorder. Biol Psychiatry. (2022) 91:498–507. doi: 10.1016/j.biopsych.2021.05.025, 34364650 PMC8636548

[9] El-Menyar A Al-Thani H Mansour MF. Dementia and traumatic brain injuries: underestimated bidirectional disorder. Front Neurol. (2024) 14:1340709. doi: 10.3389/fneur.2023.1340709, 38259659 PMC10801033

[10] Sang XZ Wang CQ Chen W Rong H Hou LJ. An exhaustive analysis of post-traumatic brain injury dementia using bibliometric methodologies. Front Neurol. (2023) 14:1165059. doi: 10.3389/fneur.2023.1165059, 37456644 PMC10345842

[11] Baethge C Goldbeck-Wood S Mertens S. SANRA-a scale for the quality assessment of narrative review articles. Res Integr Peer Rev. (2019) 4:5. doi: 10.1186/s41073-019-0064-8, 30962953 PMC6434870

[12] Schneider ALC Huie JR Boscardin WJ Nelson L Barber JK Yaffe K . Cognitive outcome 1 year after mild traumatic brain injury: results from the TRACK-TBI study. Neurology. (2022) 98:e1248–61. doi: 10.1212/WNL.0000000000200041, 35173018 PMC8967334

[13] Hammond FM Perkins SM Corrigan JD Nakase-Richardson R Brown AW O'Neil-Pirozzi TM . Functional change from five to fifteen years after traumatic brain injury. J Neurotrauma. (2021) 38:858–69. doi: 10.1089/neu.2020.7287, 32907468

[14] Nordström A Nordström P. Traumatic brain injury and the risk of dementia diagnosis: a nationwide cohort study. PLoS Med. (2018) 15:e1002496. doi: 10.1371/journal.pmed.1002496, 29381704 PMC5790223

[15] Schneider ALC Pike JR Elser H Coresh J Mosley TH Diaz-Arrastia R . Traumatic brain injury and cognitive change over 30 years among community-dwelling older adults. Alzheimers Dement. (2024) 20:6232–42. doi: 10.1002/alz.14104, 38970220 PMC11497669

[16] Lennon MJ Brooker H Creese B Thayanandan T Rigney G Aarsland D . Lifetime traumatic brain injury and cognitive domain deficits in late life: the PROTECT-TBI cohort study. J Neurotrauma. (2023) 40:1423–35. doi: 10.1089/neu.2022.0360, 36716779

[17] Brett BL Temkin N Barber JK Okonkwo DO Stein M Bodien YG . Long-term multidomain patterns of change after traumatic brain injury: a TRACK-TBI LONG study. Neurology. (2023) 101:e740–53. doi: 10.1212/WNL.0000000000207501, 37344231 PMC10437015

[18] Graham NSN Zimmerman KA Moro F Heslegrave A Abed Maillard S Bernini A . Axonal marker neurofilament light predicts long-term outcomes and progressive neurodegeneration after traumatic brain injury. Sci Transl Med. (2021) 13:eabg9922. doi: 10.1126/scitranslmed.abg9922 34586833

[19] Zanier ER Bertani I Sammali E Pischiutta F Chiaravalloti MA Vegliante G . Induction of a transmissible tau pathology by traumatic brain injury. Brain. (2018) 141:2685–99. doi: 10.1093/brain/awy193, 30084913 PMC6113646

[20] Bieniek KF Blessing MM Heckman MG Diehl NN Serie AM Paolini MA 2nd . Association between contact sports participation and chronic traumatic encephalopathy: a retrospective cohort study. Brain Pathol. (2020) 30:63–74. doi: 10.1111/bpa.12757, 31199537 PMC6916416

[21] Iliff JJ Chen MJ Plog BA Zeppenfeld DM Soltero M Yang L . Impairment of glymphatic pathway function promotes tau pathology after traumatic brain injury. J Neurosci. (2014) 34:16180–93. doi: 10.1523/JNEUROSCI.3020-14.2014, 25471560 PMC4252540

[22] Ramlackhansingh AF Brooks DJ Greenwood RJ Bose SK Turkheimer FE Kinnunen KM . Inflammation after trauma: microglial activation and traumatic brain injury. Ann Neurol. (2011) 70:374–83. doi: 10.1002/ana.22455, 21710619

[23] Johnson VE Stewart JE Begbie FD Trojanowski JQ Smith DH Stewart W. Inflammation and white matter degeneration persist for years after a single traumatic brain injury. Brain. (2013) 136:28–42. doi: 10.1093/brain/aws322, 23365092 PMC3562078

[24] Postupna N Rose SE Gibbons LE Coleman NM Hellstern LL Ritchie K . The delayed neuropathological consequences of traumatic brain injury in a community-based sample. Front Neurol. (2021) 12:624696. doi: 10.3389/fneur.2021.624696, 33796061 PMC8008107

[25] Schaffert J LoBue C White CL 3rd Chiang HS Didehbani N Lacritz L . Traumatic brain injury history is associated with an earlier age of dementia onset in autopsy-confirmed Alzheimer's disease. Neuropsychology. (2018) 32:410–6. doi: 10.1037/neu0000423, 29389151 PMC5975092

[26] LoBue C Woon FL Rossetti HC Hynan LS Hart J Cullum CM. Traumatic brain injury history and progression from mild cognitive impairment to Alzheimer disease. Neuropsychology. (2018) 32:401–9. doi: 10.1037/neu0000431, 29809031 PMC5975979

[27] Jack CR Jr Andrews JS Beach TG Buracchio T Dunn B Graf A . Revised criteria for diagnosis and staging of Alzheimer's disease: Alzheimer's Association workgroup. Alzheimers Dement. (2024) 20:5143–69. doi: 10.1002/alz.13859, 38934362 PMC11350039

[28] American Psychiatric Association. Diagnostic and Statistical Manual of Mental Disorders: DSM-5-TR. 5th ed., text revision ed. Washington, DC: American Psychiatric Association Publishing (2022).

[29] Katz DI Bernick C Dodick DW Mez J Mariani ML Adler CH . National Institute of Neurological Disorders and Stroke consensus diagnostic criteria for traumatic encephalopathy syndrome. Neurology. (2021) 96:848–63. doi: 10.1212/WNL.0000000000011850, 33722990 PMC8166432

[30] Manley GT Dams-O'Connor K Alosco ML Awwad HO Bazarian JJ Bragge P . A new characterisation of acute traumatic brain injury: the NIH-NINDS TBI classification and nomenclature initiative. Lancet Neurol. (2025) 24:512–23. doi: 10.1016/S1474-4422(25)00154-1 40409315

[31] Korley FK Yue JK Wilson DH Hrusovsky K Diaz-Arrastia R Ferguson AR . Performance evaluation of a multiplex assay for simultaneous detection of four clinically relevant traumatic brain injury biomarkers. J Neurotrauma. (2019) 36:182–7. doi: 10.1089/neu.2017.5623, 29690824 PMC6306681

[32] Devoto C Vorn R Mithani S Meier TB Lai C Broglio SP . Plasma phosphorylated tau181 as a biomarker of mild traumatic brain injury: findings from THINC and NCAA-DoD CARE consortium prospective cohorts. Front Neurol. (2023) 14:1202967. doi: 10.3389/fneur.2023.1202967, 37662031 PMC10470112

[33] Graham N Zimmerman K Heslegrave AJ Keshavan A Moro F Abed-Maillard S . Alzheimer's disease marker phospho-tau181 is not elevated in the first year after moderate-to-severe TBI. J Neurol Neurosurg Psychiatry. (2024) 95:356–9. doi: 10.1136/jnnp-2023-331854, 37833041 PMC10958285

[34] Clarke GJB Skandsen T Zetterberg H Einarsen CE Feyling C Follestad T . One-year prospective study of plasma biomarkers from CNS in patients with mild traumatic brain injury. Front Neurol. (2021) 12:643743. doi: 10.3389/fneur.2021.643743, 33967940 PMC8097004

[35] Rabinovici GD Knopman DS Arbizu J Benzinger TLS Donohoe KJ Hansson O . Updated appropriate use criteria for amyloid and tau PET: a report from the Alzheimer's Association and Society for Nuclear Medicine and Molecular Imaging Workgroup. J Nucl Med. (2025) 66:S5–S31. doi: 10.2967/jnumed.124.268756, 39778970 PMC12794165

[36] Zhang L Yang W Li X Dove A Qi X Pan KY . Association of life-course traumatic brain injury with dementia risk: a nationwide twin study. Alzheimers Dement. (2023) 19:217–25. doi: 10.1002/alz.12671, 35347847 PMC10078668

[37] Dam T Boxer AL Golbe LI Höglinger GU Morris HR Litvan I . Safety and efficacy of anti-tau monoclonal antibody gosuranemab in progressive supranuclear palsy: a phase 2, randomized, placebo-controlled trial. Nat Med. (2021) 27:1451–7. doi: 10.1038/s41591-021-01455-x, 34385707

[38] Teng E Manser PT Pickthorn K Brunstein F Blendstrup M Sanabria Bohorquez S . Safety and efficacy of semorinemab in individuals with prodromal to mild Alzheimer disease: a randomized clinical trial. JAMA Neurol. (2022) 79:758–67. doi: 10.1001/jamaneurol.2022.1375, 35696185 PMC9194753

[39] van Dyck CH Swanson CJ Aisen P Bateman RJ Chen C Gee M . Lecanemab in early Alzheimer's disease. N Engl J Med. (2023) 388:9–21. doi: 10.1056/NEJMoa2212948 36449413

[40] Sims JR Zimmer JA Evans CD Lu M Ardayfio P Sparks J . Donanemab in early symptomatic Alzheimer disease: the TRAILBLAZER-ALZ 2 randomized clinical trial. JAMA. (2023) 330:512–27. doi: 10.1001/jama.2023.13239, 37459141 PMC10352931

[41] Cummings J Apostolova L Rabinovici GD Atri A Aisen P Greenberg S . Lecanemab: appropriate use recommendations. J Prev Alzheimers Dis. (2023) 10:362–77. doi: 10.14283/jpad.2023.30, 37357276 PMC10313141

[42] Cicerone KD Goldin Y Ganci K Rosenbaum A Wethe JV Langenbahn DM . Evidence-based cognitive rehabilitation: systematic review of the literature from 2009 through 2014. Arch Phys Med Rehabil. (2019) 100:1515–33. doi: 10.1016/j.apmr.2019.02.011, 30926291

[43] Grasset L Power MC Crivello F Tzourio C Chêne G Dufouil C. How traumatic brain injury history relates to brain health MRI markers and dementia risk: findings from the 3C Dijon cohort. J Alzheimer's Dis. (2023) 92:183–93. doi: 10.3233/JAD-220658, 36710672 PMC10041415

[44] Schaffert J LoBue C Chiang HS Peters ME Hart J Jr Cullum CM. Traumatic brain injury characteristics are not related to neurocognitive decline in older adults: a nationwide longitudinal cohort study. Arch Clin Neuropsychol. (2024) 39:325–34. doi: 10.1093/arclin/acae003, 38332549 PMC11042919

[45] McKee AC Alosco ML Huber BR. Repetitive head impacts and chronic traumatic encephalopathy. Neurosurg Clin N Am. (2016) 27:529–35. doi: 10.1016/j.nec.2016.05.009, 27637402 PMC5028120

[46] Dams-O'Connor K Awwad HO Hoffman S Pugh MJ Johnson VE Keene CD . Alzheimer’s disease-related dementias summit 2022: national research priorities for the investigation of post-traumatic brain injury Alzheimer's disease and related dementias. J Neurotrauma. (2023) 40:1512–23. doi: 10.1089/neu.2022.0514, 36927167 PMC10494902

