# Supplementary material for: Post-traumatic cognitive decline and neurodegenerative disease: a narrative review and proposed framework for etiologic assessment
Source: Front Neurol. 2026 Oct 6;17:1844523. doi: 10.3389/fneur.2026.1844523 (PMC13681357; doi:10.3389/fneur.2026.1844523)
Supplement: Supplementary file 1 [file Supplementary_file_1.docx]

# Supplementary Methods S1. Literature search — execution record

This document is the execution record for the literature search described in Section 2 of the manuscript. It gives the queries as entered, the PubMed Search Details returned for each, the execution records, the result counts, the overlap accounting, and the sentinel-study check.

## S1.1 Database and scope

A de novo search of PubMed was conducted through PubMed.gov on **27 July 2026**, limited to records published from **1 January 2015** through the search date. PubMed was the only database searched at revision. The databases named in the original submission were not re-executed, and no attempt was made to reconstruct their result counts retrospectively.

Two complementary strategies were used, as described in Section 2 of the manuscript: a clinical and epidemiological search, and a biomarker and neuroimaging search.

Both queries carried the same two restrictions, visible in the strings below:

- **english[lang]** — retrieval was limited to English-language records.
- **NOT (animals[mh] NOT humans[mh])** — records indexed for animals only were excluded. Mechanistic animal work cited in this review was therefore reached by targeted retrieval and reference-list checking, not by these searches, which is what Section 2 states.

The date limit appears in the queries as 2015/01/01:2026/07/27[dp], matching the coverage stated in Section 2 from 1 January 2015 through the search date.

## S1.2 Search 1 — clinical and epidemiological

**Query as entered**

( "Brain Injuries, Traumatic"[Mesh] OR "Brain Injuries"[Mesh:noexp] OR "Brain Concussion"[Mesh] OR "traumatic brain injur*"[tiab] OR TBI[tiab] OR "head injur*"[tiab] OR "head trauma"[tiab] OR concuss*[tiab] OR "repetitive head impact*"[tiab] OR "repetitive brain trauma"[tiab] ) AND ( "Cognition Disorders"[Mesh] OR "Cognitive Dysfunction"[Mesh] OR "Dementia"[Mesh] OR "Alzheimer Disease"[Mesh] OR "Neurodegenerative Diseases"[Mesh] OR "Chronic Traumatic Encephalopathy"[Mesh] OR "Executive Function"[Mesh] OR "Activities of Daily Living"[Mesh] OR cognit*[tiab] OR neurocognit*[tiab] OR dementia[tiab] OR Alzheimer*[tiab] OR neurodegenerat*[tiab] OR "chronic traumatic encephalopathy"[tiab] OR "traumatic encephalopathy syndrome"[tiab] OR memory[tiab] OR executive[tiab] OR neuropsycholog*[tiab] OR "functional change"[tiab] OR "functional decline"[tiab] OR "functional outcome*"[tiab] OR "functional independence"[tiab] ) AND english[lang] AND 2015/01/01:2026/07/27[dp] NOT (animals[mh] NOT humans[mh])

**PubMed Search Details** (the expanded translation returned by PubMed)

(("brain injuries, traumatic"[MeSH Terms] OR "Brain Injuries"[MeSH Terms:noexp] OR "Brain Concussion"[MeSH Terms] OR "traumatic brain injur*"[Title/Abstract] OR "TBI"[Title/Abstract] OR "head injur*"[Title/Abstract] OR "head trauma"[Title/Abstract] OR "concuss*"[Title/Abstract] OR "repetitive head impact*"[Title/Abstract] OR "repetitive brain trauma"[Title/Abstract]) AND ("Cognition Disorders"[MeSH Terms] OR "Cognitive Dysfunction"[MeSH Terms] OR "Dementia"[MeSH Terms] OR "Alzheimer Disease"[MeSH Terms] OR "Neurodegenerative Diseases"[MeSH Terms] OR "Chronic Traumatic Encephalopathy"[MeSH Terms] OR "Executive Function"[MeSH Terms] OR "Activities of Daily Living"[MeSH Terms] OR "cognit*"[Title/Abstract] OR "neurocognit*"[Title/Abstract] OR "Dementia"[Title/Abstract] OR "alzheimer*"[Title/Abstract] OR "neurodegenerat*"[Title/Abstract] OR "Chronic Traumatic Encephalopathy"[Title/Abstract] OR "traumatic encephalopathy syndrome"[Title/Abstract] OR "memory"[Title/Abstract] OR "executive"[Title/Abstract] OR "neuropsycholog*"[Title/Abstract] OR "functional change"[Title/Abstract] OR "functional decline"[Title/Abstract] OR "functional outcome*"[Title/Abstract] OR "functional independence"[Title/Abstract]) AND "english"[Language] AND 2015/01/01:2026/07/27[Date - Publication]) NOT ("animals"[MeSH Terms] NOT "humans"[MeSH Terms])

**Execution:** 27 July 2026, 02:34:13. **Records returned: 16,499.**

## S1.3 Search 2 — biomarker and neuroimaging

**Query as entered**

( "Brain Injuries, Traumatic"[Mesh] OR "Brain Injuries"[Mesh:noexp] OR "Brain Concussion"[Mesh] OR "traumatic brain injur*"[tiab] OR TBI[tiab] OR "head injur*"[tiab] OR "head trauma"[tiab] OR concuss*[tiab] OR "repetitive head impact*"[tiab] OR "repetitive brain trauma"[tiab] ) AND ( "Biomarkers"[Mesh:noexp] OR "Magnetic Resonance Imaging"[Mesh] OR "Positron-Emission Tomography"[Mesh] OR biomarker*[tiab] OR "neurofilament light"[tiab] OR NfL[tiab] OR "glial fibrillary acidic protein"[tiab] OR GFAP[tiab] OR "phosphorylated tau"[tiab] OR "p-tau"[tiab] OR ptau[tiab] OR p-tau181[tiab] OR p-tau217[tiab] OR ptau181[tiab] OR ptau217[tiab] OR amyloid*[tiab] OR Abeta[tiab] OR "amyloid beta"[tiab] OR "tau PET"[tiab] OR "amyloid PET"[tiab] OR neuroimag*[tiab] OR MRI[tiab] OR "magnetic resonance imaging"[tiab] OR "positron emission tomography"[tiab] ) AND ( longitudinal[tiab] OR serial[tiab] OR follow-up[tiab] OR followup[tiab] OR "long-term"[tiab] OR chronic[tiab] OR persist*[tiab] OR delayed[tiab] OR progress*[tiab] OR prognos*[tiab] OR trajector*[tiab] OR outcome*[tiab] OR atroph*[tiab] ) AND english[lang] AND 2015/01/01:2026/07/27[dp] NOT (animals[mh] NOT humans[mh])

**PubMed Search Details**

(("brain injuries, traumatic"[MeSH Terms] OR "Brain Injuries"[MeSH Terms:noexp] OR "Brain Concussion"[MeSH Terms] OR "traumatic brain injur*"[Title/Abstract] OR "TBI"[Title/Abstract] OR "head injur*"[Title/Abstract] OR "head trauma"[Title/Abstract] OR "concuss*"[Title/Abstract] OR "repetitive head impact*"[Title/Abstract] OR "repetitive brain trauma"[Title/Abstract]) AND ("Biomarkers"[MeSH Terms:noexp] OR "Magnetic Resonance Imaging"[MeSH Terms] OR "Positron-Emission Tomography"[MeSH Terms] OR "biomarker*"[Title/Abstract] OR "neurofilament light"[Title/Abstract] OR "NfL"[Title/Abstract] OR "glial fibrillary acidic protein"[Title/Abstract] OR "GFAP"[Title/Abstract] OR "phosphorylated tau"[Title/Abstract] OR "p-tau"[Title/Abstract] OR "ptau"[Title/Abstract] OR "p-tau181"[Title/Abstract] OR "p-tau217"[Title/Abstract] OR "ptau181"[Title/Abstract] OR "ptau217"[Title/Abstract] OR "amyloid*"[Title/Abstract] OR "Abeta"[Title/Abstract] OR "amyloid beta"[Title/Abstract] OR "tau PET"[Title/Abstract] OR "amyloid PET"[Title/Abstract] OR "neuroimag*"[Title/Abstract] OR "MRI"[Title/Abstract] OR "Magnetic Resonance Imaging"[Title/Abstract] OR "Positron-Emission Tomography"[Title/Abstract]) AND ("longitudinal"[Title/Abstract] OR "serial"[Title/Abstract] OR "follow-up"[Title/Abstract] OR "followup"[Title/Abstract] OR "long-term"[Title/Abstract] OR "chronic"[Title/Abstract] OR "persist*"[Title/Abstract] OR "delayed"[Title/Abstract] OR "progress*"[Title/Abstract] OR "prognos*"[Title/Abstract] OR "trajector*"[Title/Abstract] OR "outcome*"[Title/Abstract] OR "atroph*"[Title/Abstract]) AND "english"[Language] AND 2015/01/01:2026/07/27[Date - Publication]) NOT ("animals"[MeSH Terms] NOT "humans"[MeSH Terms])

**Execution:** 27 July 2026, 02:35:25. **Records returned: 6,746.**

## S1.4 Result counts and overlap accounting

| Quantity | Unique PMIDs |
| --- | --- |
| CSV-derived set A, associated with Search 1 | 9,261 |
| CSV-derived set B, associated with Search 1 | 7,601 |
| PMIDs shared by sets A and B | 363 |
| **Search 1, unique records** | **16,499** |
| **Search 2, unique records** | **6,746** |
| PMIDs shared by Search 1 and Search 2 | 2,883 |
| Search 2 unique contribution | 3,863 |
| **Combined unique records** | **20,362** |

For record-level accounting, two CSV-derived PMID sets associated with Search 1 contained 9,261 and 7,601 records and shared 363 PMIDs. Their union contained 16,499 unique PMIDs, exactly matching the single Search 1 result count recorded in the PubMed History file. The Search 2 export contained 6,746 unique PMIDs, of which 2,883 were already present in the Search 1 union, giving a unique contribution of 3,863 and a combined total of 20,362 unique records.

Accounting: 9,261 + 7,601 − 363 = 16,499; 16,499 + 6,746 − 2,883 = 20,362.

These are retrieval counts, not screening counts. No title and abstract screening of this combined set was undertaken, and the 20,362 figure should not be read as a number of records screened or assessed for eligibility.

The CSV files themselves do not establish how the two overlapping files were generated; they are used here only for record-level set accounting.

**Record files.** Each of the three CSV exports was read as a PMID set: row counts equalled unique-PMID counts, with no blank or duplicate identifiers. NBIB exports of the same searches are incomplete (9,261 / 5,700 / 5,100 records against 9,261 / 7,601 / 6,746 actual — 3,547 records short in total) and were **not** used for record accounting.

## S1.5 Sentinel-study check

Eleven index articles were specified **before** execution and their retrieval checked afterwards. Ten of eleven were retrieved. Per-article results, and the disclosure of a PMID error made during the initial verification and corrected at source level, are in **Supplementary Table S1**.

The check was a coverage probe, not an eligibility screen. No article was included or excluded on the basis of whether a search retrieved it.

## S1.6 What this search was and was not

The searches supported targeted updating of the evidence base and verified retrieval of key literature. They were **not** an exhaustive systematic-review screening corpus. No exhaustive title/abstract screening, dual-reviewer study selection, formal risk-of-bias assessment, or quantitative synthesis was undertaken, and no PRISMA flow diagram or numerical included-study count is reported.

Diagnostic and research criteria, neuropathological reference material, mechanistic animal and smaller human studies, disease-specific comparator biomarker studies, pivotal treatment trials, and appropriate-use recommendations were identified through targeted retrieval and reference-list checking rather than through these searches.
