# Supplementary material for: Post-traumatic cognitive decline and neurodegenerative disease: a narrative review and proposed framework for etiologic assessment
Source: Front Neurol. 2026 Oct 6;17:1844523. doi: 10.3389/fneur.2026.1844523 (PMC13681357; doi:10.3389/fneur.2026.1844523)
Supplement: Supplementary file 2 [file Table_1.docx]

# Supplementary Table S1. Prespecified index articles and their retrieval status

**Search executed:** 27 July 2026, PubMed via PubMed.gov, records published 1 January 2015 through the search date.

| # | Index article | PMID | Assigned search | Retrieved |
| --- | --- | --- | --- | --- |
| 1 | Nordström & Nordström 2018 | 29381704 | Search 1 | Yes |
| 2 | Gu et al. 2022 | 34818648 | Search 1 | Yes |
| 3 | Grasset et al. 2023 | 36710672 | Search 1 | Yes |
| 4 | Schaffert et al. 2024 | 38332549 | Search 1 | Yes |
| 5 | Zhang et al. 2023 | 35347847 | Search 1 | Yes |
| 6 | Schneider et al. 2022 | 35173018 | Search 1 | Yes |
| 7 | Hammond et al. 2021 | 32907468 | Search 1 | Yes |
| 8 | Graham et al. 2021 | 34586833 | Search 2 | Yes |
| 9 | Hossain et al. 2023 | 37582732 | Search 2 | Yes |
| 10 | Shahim et al. 2017, *Neurology* **88(15)** | 28283595 | Search 2 | Yes |
| 11 | Shahim et al. 2017, *Neurology* **88(19)** | 28404801 | Search 2 | **No** |

**Retrieval:** Search 1, 7 of 7. Search 2, 3 of 4. Total **10 of 11**.

### Footnotes

**a.** Sentinel articles were used solely as probes of search coverage; inclusion in this table does not indicate citation or inclusion in the final narrative synthesis.

**b.** *Assigned search* denotes the prespecified strategy against which retrieval was evaluated and does not imply exclusive retrieval by that search. Several sentinels were returned by both searches — Grasset 2023 and Graham 2021 among them.

**c.** Rows 10 and 11 are two distinct articles published in the same volume of *Neurology* in 2017 by overlapping author groups. The journal issue is given in every mention to prevent them being read as one record.

**d.** The initial verification of row 11 used PMID 27539721 in error. Source-level verification identified the correct record as PMID **28404801**, and that article was not retrieved by either executed search. PMID 27539721 is present in the Search 2 result set but is a different article by a different first author — Ljungqvist et al., *Journal of Neurotrauma*, 2017.
